# Interocular Symmetry of Vascular Density and Association with Central Macular Thickness of Healthy Adults by Optical Coherence Tomography Angiography

**DOI:** 10.1038/s41598-017-16675-w

**Published:** 2017-11-24

**Authors:** Guodong Liu, Khusbu Keyal, Fang Wang

**Affiliations:** Department of Ophthalmology, Shanghai Tenth People’s Hospital Affliated to Tongji University School of Medicine, Shanghai, 200072 China

## Abstract

In order to analyze the interocular correlation of vascular density, foveal avascular zone (FAZ) size, central macular thickness (CMT), and to investigate the relationship between vascular density and CMT in normal eyes, retinal vascular density in superficial capillary plexus (SCP), deep capillary plexus (DCP) and choriocapillaris (CC) networks, FAZ size, and CMT were visualized by optical coherence tomography (OCT) angiography. A total of 174 eyes of 87 normal Chinese subjects were enrolled in the study. The results showed that FAZ and CMT measurements are not statistically significant between right and left eyes, but right eyes had higher vascular density in superficial, deep retinal capillary and choriocapillaris networks, which might be related with dominant eyes. Spearman correlation test revealed a high correlation between right and left eyes for FAZ and CMT measurements (r = 0.934; r = 0.935), and a moderate correlation for SCP, DCP and CC density (r = 0.402; r = 0.666; r = 0.417). The analysis showed greater vascular density with smaller FAZ size, and a high negative relationship between FAZ and CMT, which indicates a positive correlation between retinal blood supply and retinal thickness.

## Introduction

Retina is one of the highest metabolic tissues in human body with great vascular density. The histologic findings identify at least two retinal capillary plexuses within macular area: the superficial capillary plexus (SCP) within the nerve fiber layer and the ganglion cell layer; deep capillary plexus (DCP) between inner nuclear layer and outer plexiform layer^1,2^. Normal vascular system of retina and choriocapillaris is vital for maintaining visual function and the changes of vascular system is important to assess the retinal and choroidal diseases, so it is necessary to evaluate vascular features of retina and choroid. Nowadays, fluorescein angiography combined with indocyanine green angiography (ICGA) is the main method to assess vascular system abnormality such as diabetic retinopathy, retinal vein occlusion, and choroidal neovascularization. However, because of the vascular plexuses overlaying and fluorescein leakage, fluorescein angiography is not able to show capillary details, especially in deep retinal layers.

Recently, split-spectrum amplitude-decorrelation angiography (SSADA) associated with optical coherence tomography (OCT) angiography (Optovue, Inc., Fremont, CA, USA) provides a novel method for noninvasively imaging the capillary network and foveal avascular zone (FAZ) without dye injection^3,4^. AngioAnalytics incorporated into the AngioVue OCT can automatically quantify nonflow area and vessel density of retinal and choroidal complexes: SCP, DCP, and choriocapillaris (CC)^5^. Because of the safety and convenience, OCT angiography has been used to investigate various retinal vascular pathologies such as macular telangiectasia^6^, retinal vein occlusion^7^, diabetic retinopathy^8^ and choroidal neovascularization^9^. The results show that OCT angiography allows better visualization of nonperfusion area, morphology of retinal vessels, and neovascularization.

In addition to the vascular changes of retinal diseases, studies also focused on the normative data for the measurements of vascular density, FAZ size and radial peripapillary capillary density in normal eyes^4,5,10^. However, the results of the studies are discrepant, mainly caused by different OCT devices, analysis software, sample size and the criterion for enrollment. Thus, a more accurate and standard method to evaluate the macular measurements in a larger sample is needed. Sometimes, we want to assess the changes of vascular density after operation to explain some phenomena, such as poor vision recovery. But it is difficult to measure the preoperative vascular density in surgery eyes, such as epi-retinal membranes and retinal detachment. So we cannot compare the vascular changes pre and post operation. Hence, it is necessary to study the interocular symmetry of macular vascular measurements in normal subjects, as it can be used as a reference value to assess the preoperative macular measurements of operated eyes according to the fellow eyes. But the published literature has not revealed any results concerning the interocular symmetry of vascular density, FAZ size, and relationship with CMT in healthy subjects.

Therefore, the aim of our study is to quantify vascular density, the FAZ area and central foveal thickness by OCT angiography automatically, and to analyze the differences of the macular measurements between right and left eyes. Next, we will furtherly evaluate the relationship between vascular density and macular retinal thickness in healthy subjects.

## Results

A total of 174 eyes of 87 normal Chinese subjects (86 eyes of 43 males, 49.4%; 88 eyes of 44 females, 50.6%) were included in this study. The mean age of the participants was 38.7 ± 15.9 years (range, 20–80 years), of which 56 eyes were in group 1 (20–30 years old), 72 eyes in group 2 (30–50 years old), and 46 eyes in group 3 (50 years and older). In 79 subjects (90.8%) the right eye was the dominant eye, and in 8 subjects (9.2%) the dominant eye was left eye.

### Quantification of FAZ area

**Figure 1:**
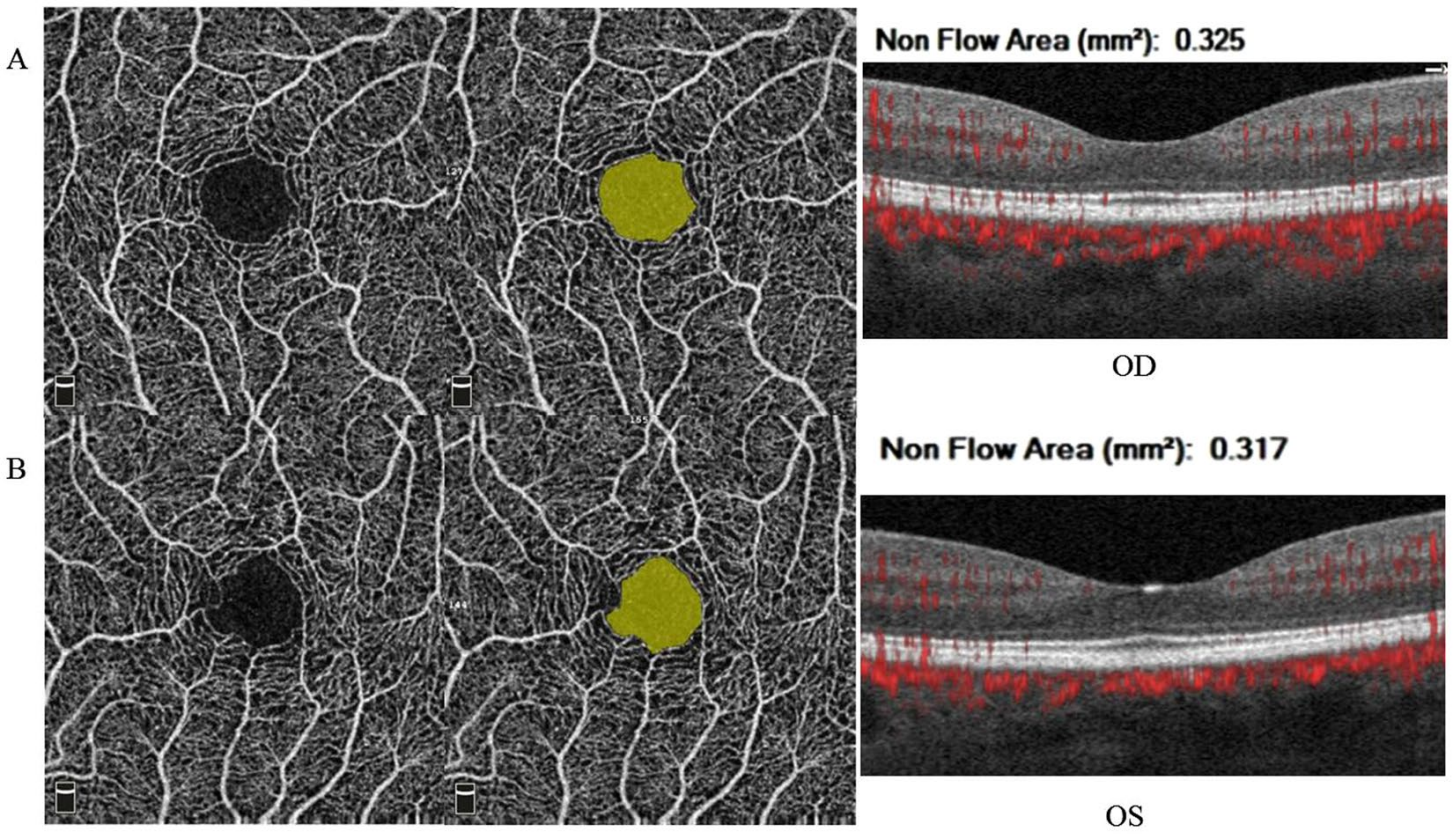
Quantification of FAZ area. The foveal avascular zone was selected at the superficial capillary plexus by AngioAnalytics. The FAZ area was graphically highlighted and calculated automatically. In this subject, the FAZ area was 0.325 mm^2^ in right eye and 0.317 mm^2^ in left eye. The FAZ values and morphology of the area were similar in both eyes.

From the Fig. 1, we know that OCT angiography can visualize the FAZ morphology and FAZ size accurately. The morphology of the FAZ varies from large, regular, circular shapes to small, irregular structures with bridging vessels, and the area measurements were from 0.042 mm^2^ to 0.738 mm^2^. But in terms of a single individual, shape and size of the FAZ are similar between right eye and left eye. In our study, the mean FAZ area in right eyes was 0.33 ± 0.11 mm^2^, while in left eyes was 0.33 ± 0.12 mm^2^ in the superficial retinal vascular layer.

### Measurements of vascular density and CMT

**Figure 2:**
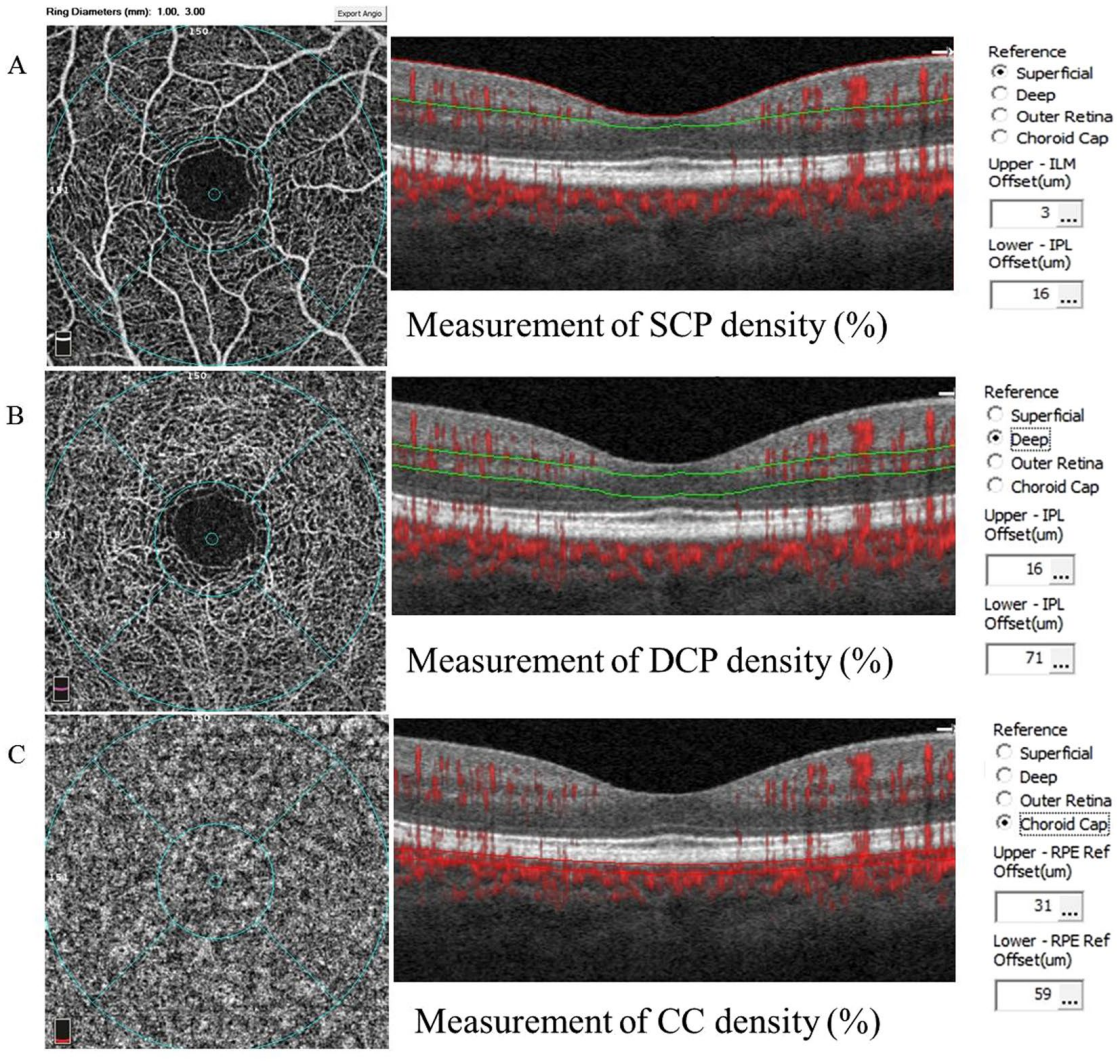
Methodology for quantification of vascular density. OCT angiography represents the superficial, deep retinal capillary network and choriocapillaris network. (**A**) The superficial retinal capillary extending from the internal limiting membranes with an offset of 3 um to the inner plexiform layer (IPL) with offset of 16 um. (**B**) The deep retinal capillary from the IPL with an offset of 16 um to 71 um. (**C**) The choriocapillary from RPE with an offset of 31 um to the choroidal layer with an offset of 59 um.

Figure 2 shows the methodologies of density quantification in different vascular layers. The vascular density in superficial and deep retinal capillary network was 53.01 ± 2.65% and 58.17 ± 2.75%, the density of choriocapillaris network was 66.13 ± 1.46%, and CMT was 241.47 ± 21.76um in right eyes, while data in left eyes was 52.09 ± 2.45%, 57.24 ± 2.75%, 65.54 ± 1.61%, and 241.40 ± 21.96um, respectively.

### Relationship between right and left eyes for macular measurements

The difference of FAZ and CMT measurements between right and left eyes were not statistically significant, but right eyes had a higher vascular density in superficial and deep retinal capillary network as well as choriocapillary network. However, 8 individuals with left dominant eyes maintain opposite results with higher retinal vascular density in left eyes except for choriocapillary density. The results imply that the higher vascular density might be related with dominant eyes. The detailed information of statistical analysis was summarized in Tables 1 and 2.

**Table 1:** Values of macular measurement between right and left eyes in 87 healthy individuals

	OD	OS	T	P
FAZ (mm^2^)	0.33 ± 0.11 (0.042–0.738)	0.33 ± 0.12 (0.031–0.745)	−0.125	0.901
SCP density (%)	53.01 ± 2.65 (43.13–57.12)	52.09 ± 2.45 (44.44–57.03)	2.83	0.005
DCP density (%)	58.17 ± 2.75 (47.09–63.2)	57.24 ± 2.75 (46.25–62.21)	2.38	0.017
CC density (%)	66.13 ± 1.46 (61.46–68.97)	65.54 ± 1.61 (56.37–68.87)	2.42	0.016
CMT (um)	241.47 ± 21.76 (196–333)	241.40 ± 21.96 (195–330)	−0.42	0.678

FAZ = foveal avascular zone, SCP = superficial capillary plexus, DCP = deep capillary plexus, CC = choriocapillaris, CMT = central macular thickness.

**Table 2:** Values of macular measurement in 5 individuals with left dominant eyes.

Patients	SCP density (%)		DCP density (%)		CC density (%)	
	OD	OS	OD	OS	OD	OS
Patient 1	50.81	51.26	55.44	56.62	64.92	66.75
Patient 2	51.63	53.29	54.02	56.10	66.29	67.03
Patient 3	51.63	56.17	54.94	60.20	70.34	67.82
Patient 4	52.17	53.41	58.97	60.15	65.12	68.51
Patient 5	50.29	53.69	55.54	58.04	63.91	67.38
Patient 6	55.37	56.31	60.62	61.12	67.28	67.26
Patient 7	52.36	55.72	56.93	60.72	65.22	65.07
Patient 8	53.24	54.29	54.98	59.06	66.05	65.74

SCP = superficial capillary plexus, DCP = deep capillary plexus, CC = choriocapillaris.

**Figure 3:**
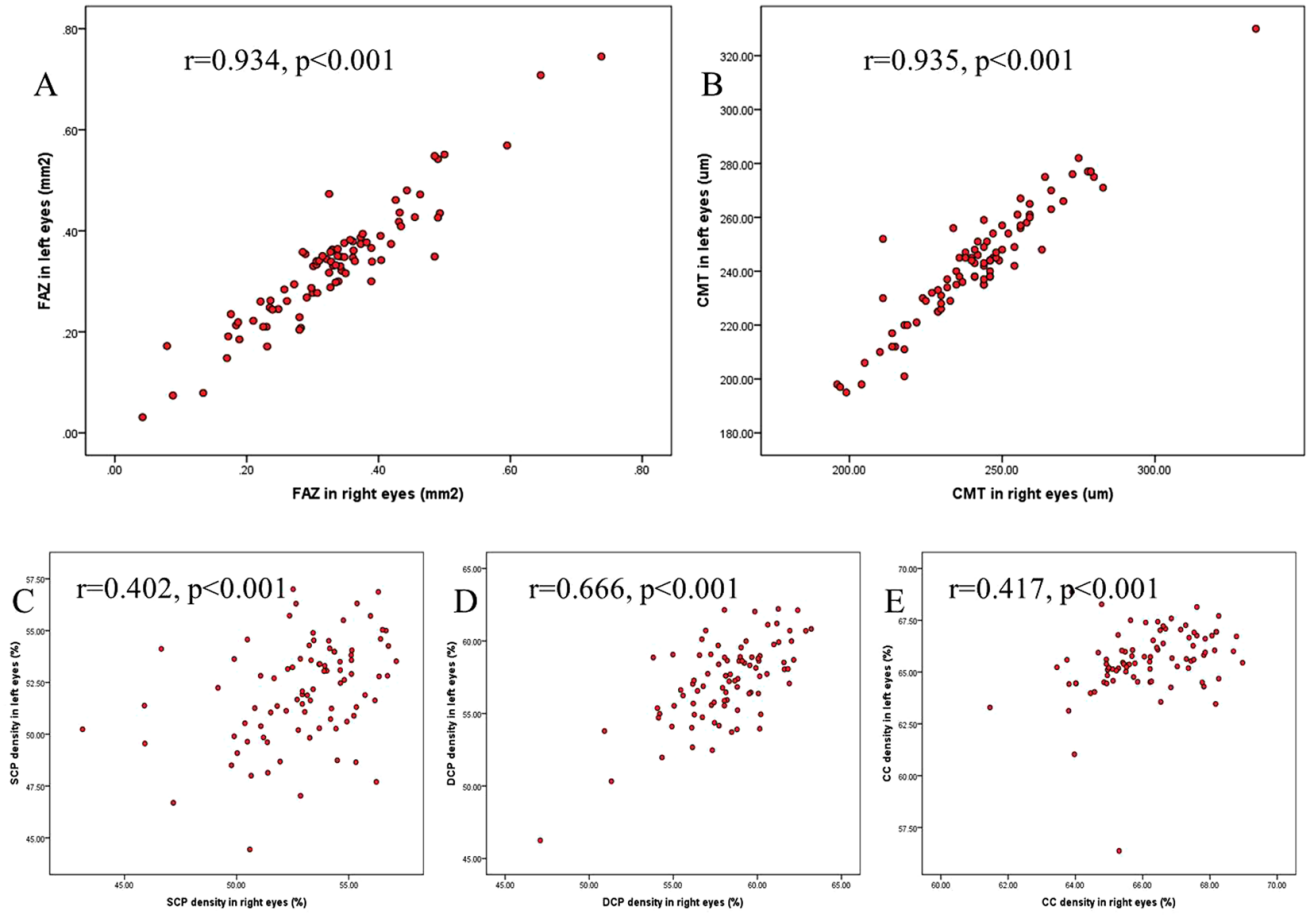
Relationship between right and left eyes for macular measurements. The red scatterplots present the relationship between right and left eyes for FAZ, CMT, SCP, DCP and CC measurements. A significant correlation was present in the FAZ area (**A**) and CMT (**B**) (r = 0.934, p < 0.001; r = 0.935, p < 0.001). Similarly, a moderate correlation was present in superficial (**C**), deep (**D**) retinal capillary network and choriocapillaris network (**E**) (r = 0.402, p < 0.001; r = 0.666, p < 0.001; r = 0.417, p < 0.001).

Spearman correlation test showed a high correlation between right and left eyes for FAZ and CMT measurements (r = 0.934, p < 0.001; r = 0.935, p < 0.001) (Fig. 3), and moderate correlations for SCP, DCP, and CC density (r = 0.402, p < 0.001; r = 0.666, p < 0.001; r = 0.417, p < 0.001) in our study (Fig. 3). So the correlation analysis indicates high interocular symmetry relationship for macular measurements, especially for FAZ and CMT values.

### Correlation between CMT and FAZ area

The results showed a high symmetrical relationship for FAZ and CMT measurements between right and left eyes, thus we supposed an area-thickness relationship might exist between FAZ and CMT. Results of Spearman correlation test showed a strong negative correlation between FAZ and CMT values (r = −0.712, p < 0.001) (Fig. 3), which suggests that eyes with smaller FAZ area had greater CMT and eyes with larger FAZ area had smaller CMT. But when the value of CMT was approximately 200um, the thickness was maintained stable, regardless of the increased FAZ size in 10 eyes.

**Figure 4:**
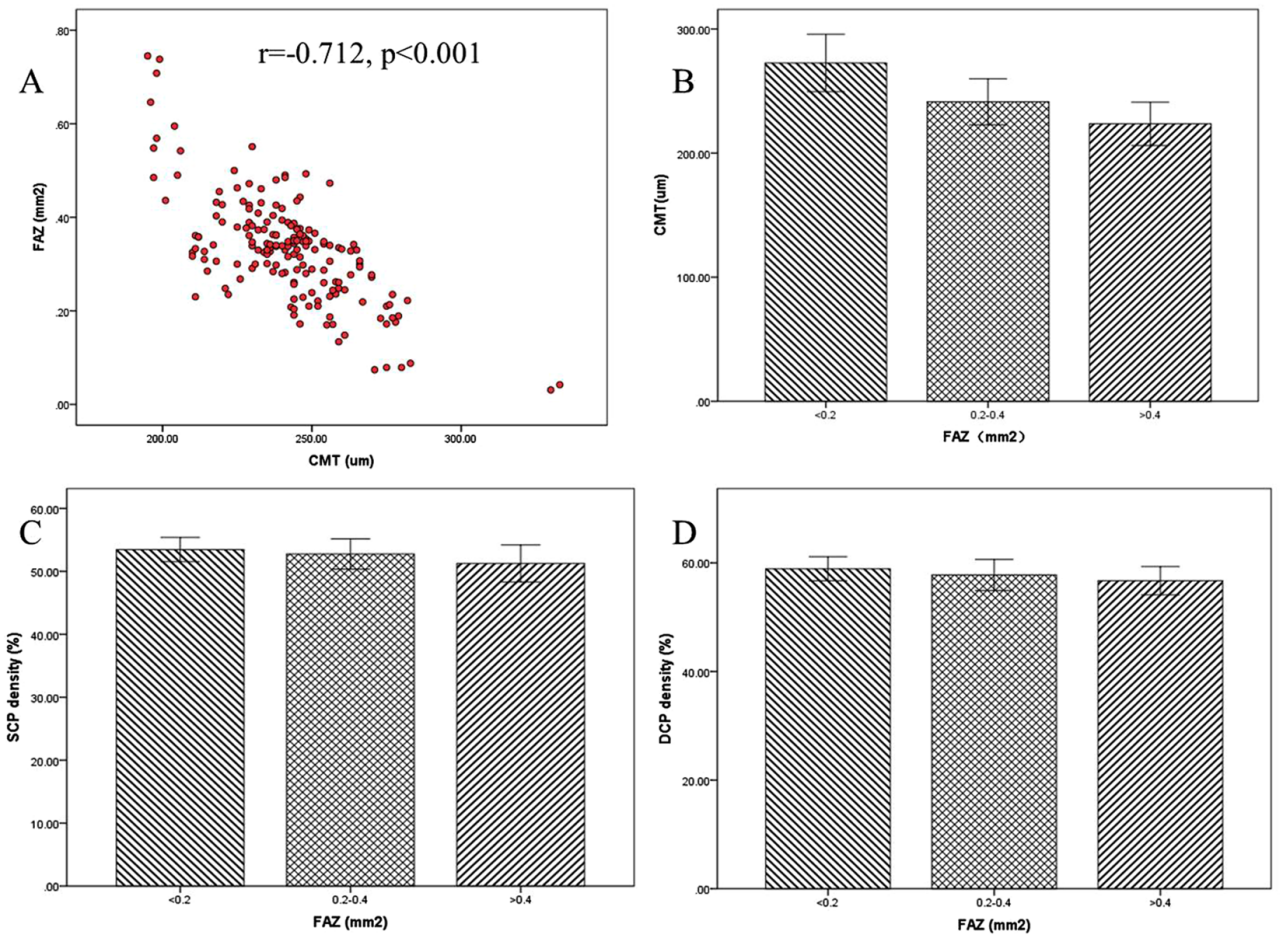
Correlation between CMT and FAZ area. The red scatterplots show a significant negative correlation between FAZ area and CMT using Spearman correlation test (r = −0.712, p < 0.001) (**A**). But when CMT closes to 200um, CMT can maintain a stable thickness, regardless of the increased FAZ size. The results of three groups with different FAZ area revealed that the eyes with smaller FAZ showed thicker CMT (p < 0.001) (**B**), greater SCP density (p < 0.01) (**C**) and DCP density (p < 0.01) (**D**).

According to the FAZ area (0.042–0.738 mm^2^), the subjects were divided into three groups, of which 22 eyes were in group 1 (<0.2 mm^2^), 112 eyes in group 2 (0.2–0.4 mm^2^), and 40 eyes in group 3 (>0.4 mm^2^). The results showed that eyes with smaller FAZ area had thicker CMT (p < 0.001) and greater retinal capillary networks (p < 0.01) (Fig. 4). The results suggest a positive relationship between blood supply and retina thickness.

### Correlation between choriocapillaris density and FAZ area

**Figure 5:**
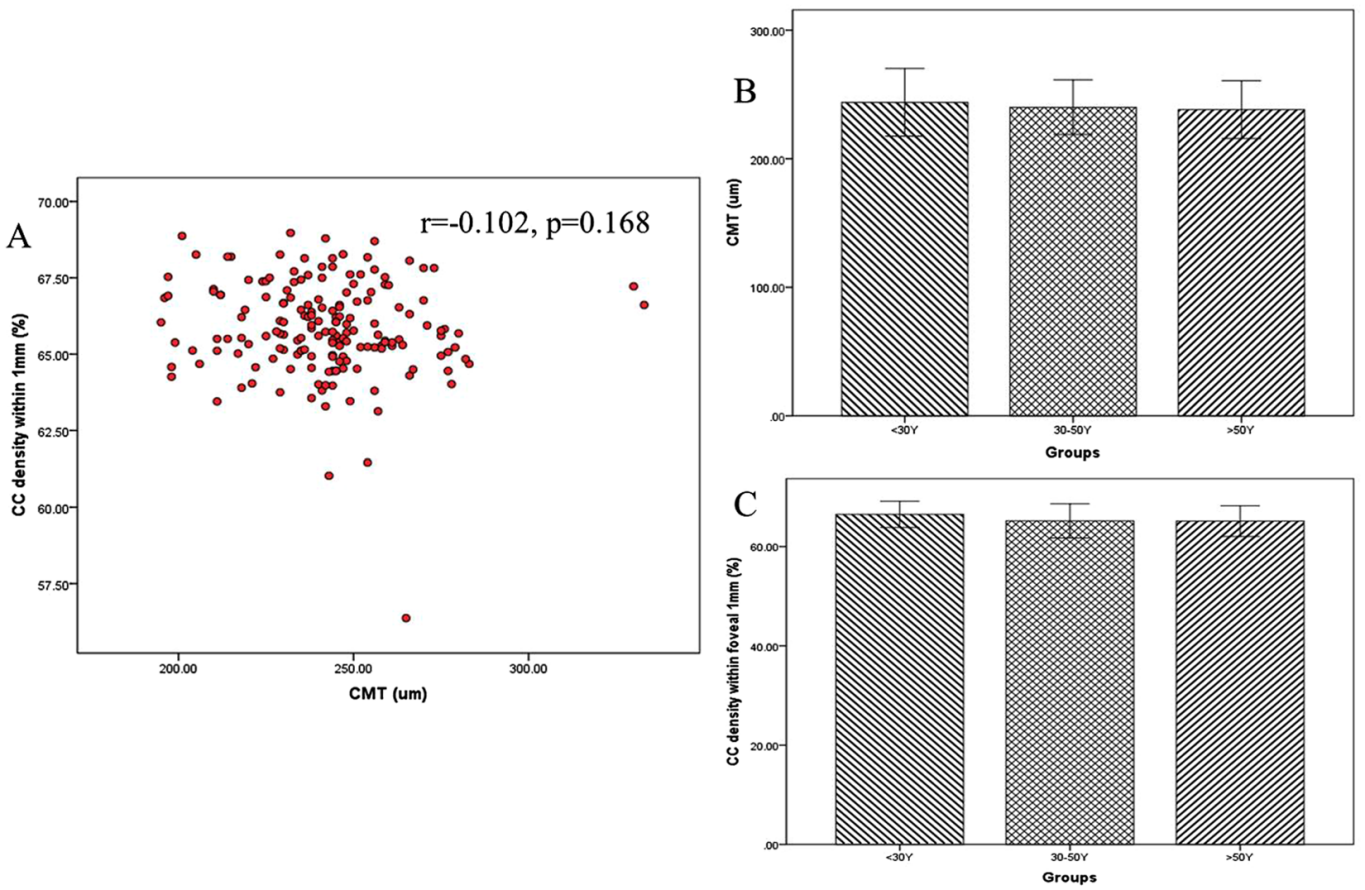
Correlation between choriocapillaris density and CMT. (**A**) The analysis did not show a significant correlation between choriocapillaris density and CMT (r = −0.102, p = 0.168). According to the age, the subjects were divided into three groups. But the values of CMT (p > 0.05) (**B**) and choriocapillaris density (p > 0.05) (**C**) has no significant difference among three groups.

Since the outer retinal layers are mainly supplied by choroid vessels, we suppose a vascular-structural relationship between choriocapillaris density and CMT. However, the correlation analysis showed no significant correlation between the two parameters (r = −0.102, p = 0.168) (Fig. 5). Initially, we considered that the changes of CMT and choriocapillaris density are variable by age growth. According to the age, the subjects were divided into three groups, of which 56 eyes were in group 1 (20–30 years old), 72 eyes in group 2 (30–50 years old), and 46 eyes in group 3 (50 years and older), then detected the difference of CMT and choriocapillaris density. But the values of this two measurements are insignificantly different in the three groups (p > 0.05) (Fig. 5).

## Discussion

OCT angiography is a noninvasive imaging technique that can visualize blood vessels and nonperfusion area without dye injections, which can also provide detailed information of vascular density and FAZ size. So OCT angiography has been used to qualify microvascular changes in many ocular diseases, such as diabetic retinopathy^11^, peripapillary retinal perfusion in glaucomatous eyes^12^ and anterior ischemic optic neuropathy (AION) eyes^13^. Recently, several studies report the normative data of vascular density and FAZ area by OCT angiography in normal subjects^14–16^. However the results demonstrated inconsistent results: FAZ size (0.28 ± 0.10 mm^2^ to 0.35 ± 0.12 mm^2^), superficial retinal vessel density (17.21 ± 3.09% to 52.58 ± 3.22%), deep retinal vessel density (34.81 ± 5.28% to 57.87 ± 2.82%), and choriocapillaris density (44.5% ± 2.7% to 62.34 ± 1.05%)^4,5,17^. The reasons for the incompatible values might be the different OCT angiography devices, inconsistent measure method and variable sample size.

Recently, a novel software program of AngioAnalytics incorporated into the AngioVue system enables the quantitative evaluation of FAZ area and vessel density in different layers automatically. The study by Coscas F used AngioAnalytics to measure the macular values, mean area of the FAZ was 0.27 ± 0.10 mm^2^, the vessel density in superficial and deep retinal capillary plexuses was 52.58 ± 3.22% and 57.87 ± 2.82% in 34 eyes of 17 healthy subjects^5^. These values are similar with the results in our study, FAZ area 0.33 ± 0.12 mm^2^, superficial and deep retinal capillary plexuses 52.55 ± 2.58% and 57.70 ± 2.78%, respectively. But, our study has bigger sample size compared to previous study and we further analyzed the symmetrical relationship of the binocular measurements and the relationship between vascular density and structural thickness.

Clinically, the relationship of vascular density and macular thickness is important between right eye and left eye. As it is difficult to measure the preoperative vascular density accurately in some retinal diseases because of abnormal retinal structure, so we cannot assess the vascular density changes after the operation. Thus, we cannot assess the effects of gas filling and surgical management on blood supply status to explain some phenomena, such as poor visual acuity with recovered retina structure. However, while knowing the interocular symmetry relationship, we can evaluate the changes based on the fellow eyes. Hence, it is necessary to study the interocular symmetry of macular vascular measurements in cases of uniocular or asymmetrical macular involvement.

In the present study, we revealed that CMT and FAZ area values were similar with no significant difference between right and left eyes. And Spearman correlation test showed a strong correlation between right and left eyes for the two measurements. But quantitative analysis of the macular measurements discloses a higher vascular density of SCP, DCP, and CC in right eyes. And Spearman correlation test showed a moderate correlation for the measurements between the right and left eyes.

The reasons for the moderate correlation of vascular density are mainly different quality signal strength index [SSI], and the complexity of vascular networks. While it is easier to define outlines of FAZ area and boundaries of CMT between inner limiting membrane and RPE layer, accurate measurements of FAZ area and CMT with high reproducibility and reliability can be acquired^18,19^, so while a high interocular symmetry exits, the relationship of the measurements can be obtained in the present study. However, it is more complex to quantify vascular density, especially in deep vascular layers. Sometimes it is inappropriate to detect vascular density with the fixed measurement model in some individuals. Besides, the measurements are more easily influenced by shadow artifacts, eye movement and different quality SSI between left and right eyes, particularly in aging patient with media opacity. Although we try our best to acquire the clearest macular scans and guarantee the SSI higher than 60, it is difficult to maintain the same SSI between right and left eye. Because of the complexity of vascular networks, it is common to get some variations of vascular networks between the right and left eyes. Those might be the reasons accounting for the moderate correlations of vessel density in different vascular layers.

The higher vascular density in right eyes is supposed to be associated with the dominant eyes. So we furtherly investigated the dominant eyes among the participators, as expected, most individual are right dominant eyes. While some subjects with left dominant eye showed higher retinal vascular density in left eyes regardless of the overall results, but the results did not show the stable trend in choriocapillaris density measurements. But the sample size of left dominant eye is small, further studies with larger sample size are needed to confirm the conclusion.

Previous studies report the correlation between FAZ area and CMT in healthy subjects and diabetic retinopathy eyes^20,21^, but we investigate the correlation in a greater sample size and relationship with vascular density measurements. Spearman correlation test showed a significant negative correlation between FAZ and CMT (r = −0.712, p < 0.001), indicating that CMT decreases with the increased FAZ area in normal subjects. But the CMT can maintain a stable thickness, when the values close to 200um, regardless of the increased FAZ area in some eyes. According to the FAZ area, CMT and vascular density were divided into three groups. The results showed that the eyes with smaller FAZ displayed greater vascular density and thicker CMT, while the eyes with larger FAZ showed smaller vascular density and thinner CMT. So there is a high negative relationship between FAZ and vascular density as well as CMT. As the higher vascular density and smaller FAZ suggest greater blood supply, the result suggests a positive correlation between blood supply and structural thickness.

In the central retinal artery occlusion (CRAO) patients, retina shows greater inner retinal thickening than outer retina^22,23^. The changes suggest that the metabolic exchanges of inner retina are highly dependent on the retinal flow, while outer retina is mainly supplied by the choriocapillaris, and only 15% of the oxygen supply is provided by the deep capillary plexuses^24^. So the FAZ area without retinal vessels is supposed to be related with choriocapillaris density closely, and a significant relationship between CMT and choriocapillaris density might exist. Unexpectedly, no relationship was found between choriocapillaris density and CMT in our study. The reasons for the result might be that the macular retinal thickness is not significantly variable in different age groups^25^, while the thickness of choroid becomes thinned with increasing age^26^. Thus, visualization and quantification of choriocapillaris density in different age groups are of great interest. But our study did not identify any flow alterations in the choriocapillaris density and CMT measurements among different age groups. Thus the large and middle choroidal vessels thinning mainly contribute to the reduced choroidal thickness without significant choriocapillary changes. But previous study confirms that the FAZ size increases and retinal vascular density decreases with increasing age^27^. While FAZ size is correlated with CMT closely, the results of increased FAZ size with insignificant CMT and unchanged choriocapillaris density in aging group need further studies.

In our study, the development of OCT angiography is a useful instrument to visualize the non-perfusion area as well as the superficial, deep retinal capillary plexuses and choriocapillaris network. We observe the histological correlation between foveal thickness and FAZ area as well as retinal vascular plexus density that smaller FAZ area is associated with thicker CMT and greater retinal vascular density. Thus, accurate and standardized evaluation of macular and choriocapillary density by OCT angiography is helpful to achieve a better understanding of the characteristics of macular blood supply and the relationship with structural thickness.

Nevertheless, this study has its limitations: although the SSI of all the subjects are no less than 60, it should be noted that SSI is higher in younger eyes compared with older eyes, because of the mild cataract, vitreous liquefaction, and other media opacities. Further study will be performed to confirm the effects of different SSI levels on vascular density by a larger sample size.

In conclusion, our study demonstrates that OCT angiography has the capability to visualize FAZ area and the capillary plexuses in different vascular layers precisely, as well as useful quantitative information. In addition, we used the device to confirm the interocular symmetry of the macular vascular density between right and left eyes and association with central macular thickness in healthy subjects.

## Methods

### Study design and subjects

From February 2017 to July 2017, 87 volunteers with best-corrected visual acuity (BCVA) of 20/20 or better and ametropic of less than 3 diopters in both eyes were enrolled in this study. The subjects have no history or clinical evidence of any retinal diseases and without any systemic diseases such as diabetes, high blood pressure or cardiovascular diseases. The individuals with any use of systemic medicines that affect the blood flow are also excluded in the study. Informed consent was obtained from all participants. Procedures followed the tenets of the Declaration of Helsinki, and this study was approved by the institutional review board of Shanghai Tenth People’s Hospital.

All subjects underwent a complete ophthalmic examination including BCVA, slit lamp biomicroscopy, intraocular pressure, dilated fundus examination, and OCT angiography examinations. Both eyes of the participants were scanned by an experienced doctor during the same visit. Exclusion criteria were any history or any signs of chorioretinal disease, glaucoma, and ocular surgery. Images with quality signal strength index [SSI] lower than 60 were excluded due to media opacities. In-cooperative patients were also excluded as it influences the sharpness of vessels. A 3 × 3 mm area, centered on the fovea, was obtained including one horizontal priority and one vertical priority in both eyes for the subjects consecutively. The detailed information of OCT angiography including SSADA method has been described in previous articles^3,5^. Image analysis was performed by automated retinal segmentation which segments the tissue into 3 layers: superficial retinal capillary plexus starting with the inner limiting membrane with an offset of 3 um to the inner plexiform layer with an offset of 16 um; the deep retinal capillary plexus from inner plexiform layer with an offset of 16 um to the inner plexiform layer with an offset of 71 um; and the choriocapillaris layer from the RPE with an offset of 31 um to the deeper choroidal layer with an offset of 59 um in this machine. The motion artifacts were decreased by eye tracking mode and removed by motion correction technology.

In order to evaluate the effect of age on the CC measurements, the study subjects were divided into three age groups: group 1 ranged from 20 to 30 years of age, group 2 ranged from 30 to 50 years, and group 3 were 50 years or older.

### Measurement of vascular density

OCT angiography was examined with the spectral domain system RTVue-XR Avanti (Optovue Inc. Fremont, CA). Vascular density was defined as the proportion of the total area occupied by vessels. The inner and outer rings with a diameter of 1.0 and 3.0 mm around the fovea were evaluated, respectively. In the present study, the flow density map software AngioAnalytics (version 2016.1.0.26) was used to quantify nonflow area and vessel density automatically. The density of SCP, DCP and CC were quantified by the superficial pattern, Deep pattern and Choroid Cap pattern automatically.

### Measurement of FAZ area

An avascular area is defined by borders of the vascular area, and the area measurement at the level of the superficial capillary plexuses was quantified by the nonflow area pattern. While the observers click on the center of the avascular area, size of the nonflow area can be showed automatically. CMT is defined as the average thickness in the central 1 mm subfield centered on the fovea.

### Dominant eye detection

All the subjects are asked to detect dominant eye by the hole-between-hands test. The subjects were guided to cross hands and to view a 5-m target through the middle space between the crossing hands. By covering each eye alternately, the observers were asked that which eye visualized the same target consistent with the both eyes. The selected eye is the dominant eye.

### Data Availability

The datasets generated and/or analyzed during the current study are available from the corresponding author on reasonable request.

### Statistical Analysis

The statistical analysis was performed with the Statistical Package for the Social Sciences (SPSS) for Windows (version 20.0; SPSS Inc.; Chicago, IL). Differences of FAZ area, vascular density and CMT were tested by the Mann–Whitney U test and One-way ANOVA. Associations between FAZ area, vascular density and CMT were analyzed using Spearman rank correlation. Values were presented as the means ± standard deviation, and criterion significance was assessed at the P < 0.05 level.

### Ethical approval and informed consent

Procedures followed the tenets of the Declaration of Helsinki, and this study was approved by the institutional review board of Shanghai Tenth People’s Hospital. Principles of human subject research were conducted in accordance with the Declaration of Helsinki. Informed consent was obtained from all participants.
